# Roles of Key Epigenetic Regulators in the Gene Transcription and Progression of Prostate Cancer

**DOI:** 10.3389/fmolb.2021.743376

**Published:** 2021-12-15

**Authors:** Tanggang Deng, Yugang Xiao, Yi Dai, Lin Xie, Xiong Li

**Affiliations:** ^1^ Key Specialty of Clinical Pharmacy, The First Affiliated Hospital of Guangdong Pharmaceutical University, Guangzhou, China; ^2^ NMPA Key Laboratory for Technology Research and Evaluation of Pharmacovigilance, Guangdong Pharmaceutical University, Guangzhou, China; ^3^ School of Clinical Pharmacy, Guangdong Pharmaceutical University, Guangzhou, China

**Keywords:** prostate cancer, androgen receptor, epigenetic regulators, diagnostic markers, therapeutic targets

## Abstract

Prostate cancer (PCa) is a top-incidence malignancy, and the second most common cause of death amongst American men and the fifth leading cause of cancer death in men around the world. Androgen receptor (AR), the key transcription factor, is critical for the progression of PCa by regulating a series of target genes by androgen stimulation. A number of co-regulators of AR, including co-activators or co-repressors, have been implicated in AR-mediated gene transcription and PCa progression. Epigenetic regulators, by modifying chromatin integrity and accessibility for transcription regulation without altering DNA sequences, influence the transcriptional activity of AR and further regulate the gene expression of AR target genes in determining cell fate, PCa progression and therapeutic response. In this review, we summarized the structural interaction of AR and epigenetic regulators including histone or DNA methylation, histone acetylation or non-coding RNA, and functional synergy in PCa progression. Importantly, epigenetic regulators have been validated as diagnostic markers and therapeutic targets. A series of epigenetic target drugs have been developed, and have demonstrated the potential to treat PCa alone or in combination with antiandrogens.

## Introduction

Prostate cancer (PCa) is the second most commonly diagnosed cancer type and the fifth leading cause of cancer death in men around the world (Bray et al., 2018). Perturbed transcriptional control is one of main mechanisms driving the development of PCa. Multiple key transcription factors (TFs) crucial for PCa progression have been identified, such as androgen receptor (AR), FOXA1, N-myc and the ETS-domain transcription factor family, etc. AR is the most important TF promoting the progression of hormone-dependent and independent PCa. Since the androgen/AR axis plays key roles in driving PCa progression, androgen deprivation therapy (ADT) remains the mainstream therapeutic modality for PCa (Kawakami et al., 2006). However, most PCa patients eventually become refractory to ADT, and the disease inevitably develops to castration-resistant prostate cancer (CRPC). At this stage, bone metastasis develops, the life quality of patients deteriorates, and commonly the life span is less than 1 year (Kirby et al., 2011). Thus, it remains urgent to clarify the molecular mechanism of CRPC and identify new diagnostic and therapeutic targets, so PCa patients can be diagnosed sooner and effectively treated.

Epigenetics refers to biological processes that regulate gene expression and function without altering DNA sequences (Holliday, 1994; Weinhold, 2006). Epigenetic modifications, including DNA methylation, histone modifications and non-coding RNA (Liao and Xu, 2019), regulate gene transcription, including the addition of chemical “tags” on DNA and the posttranslational modification (PTM) of histone proteins, imparting distinct features on chromatin architecture. Currently the key enzymes catalyzing these modifications have been identified as epigenetic regulators and categorized as “writers”, “readers” and “erasers”. “Writers” such as DNA methyltransferases (DNMTs) and histone acetyltransferases (HATs) introduce chemical modifications such as methylation and/or acetylation on DNA and histone proteins. “Readers” are the specialized proteins that identify and interpret those modifications and convey the epigenetic information to downstream effectors. “Erasers” are enzymes proficient in removing these epigenetic markers, and include histone demethylases (KDMs) and histone deacetylase complexes (HDACs) (Yegnasubramanian, 2016).

These epigenetic modifications enhance the establishment of a context-specific transcriptional profile, and aberrations ultimately cause genomic instability. Aberrations of epigenetic regulators such as gene amplification or mutations have been frequently found in PCa (Li et al., 2019), and play vital roles in the initiation and progression of cancer (Graca et al., 2016). The epigenetic regulators, individually or cooperatively with AR, contribute PCa progression as transcriptional co-activators or co-repressors of AR.

In this review, we summarized the known epigenetic regulators, including readers, writers, and erasers working together with AR in PCa progression, and discussed their potential as epigenetic diagnostic and therapeutic targets, as well as future translational applications for PCa.

## Androgen/Androgen Receptor Pathways and Epigenetic Co-regulators in Prostate Cancer

**FIGURE 1:**
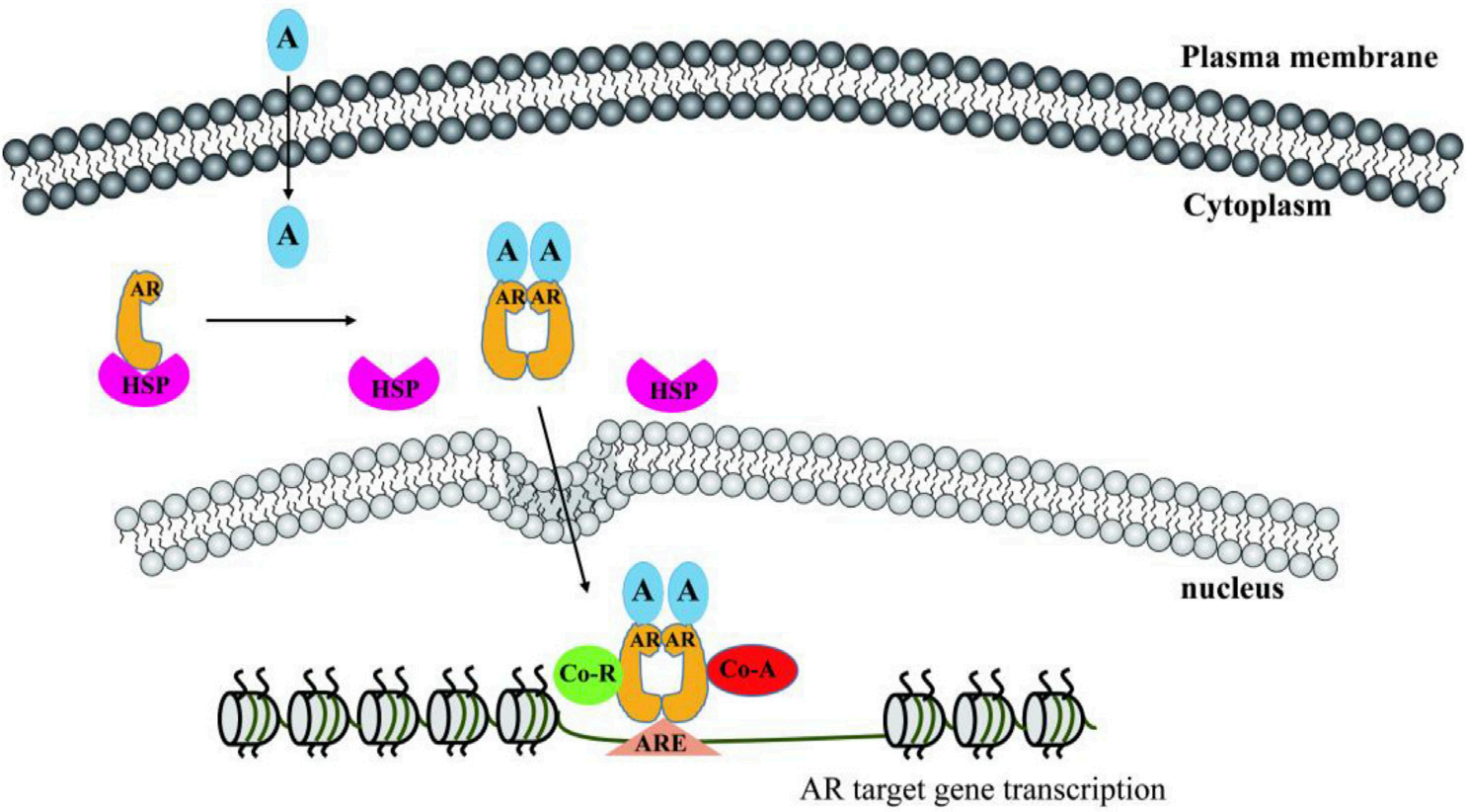
Androgen/AR signaling pathway. A, androgen; AR, androgen receptor; HSP, heat shock protein; ARE, androgen response element; Co-R, co-repressor; Co-A, co-activator.

Androgen is mainly produced by the conversion of cholesterol in the testes, muscle, adrenal glands, ovaries, skin, adipose tissue and endometrium (Narayanan et al., 2018). Androgen is a major male steroid that binds to AR to determine masculinity and sexual maturation. AR dissociates from heat shock proteins (HSPs), and AR phosphorylation promotes AR translocation to the nuclei where AR binds to the androgen response element (ARE) motifs through zinc finger domains. AR recruits co-regulators, and regulates the transcription of downstream target genes (Eftekharzadeh et al., 2019) (Figure 1).

**FIGURE 2:**
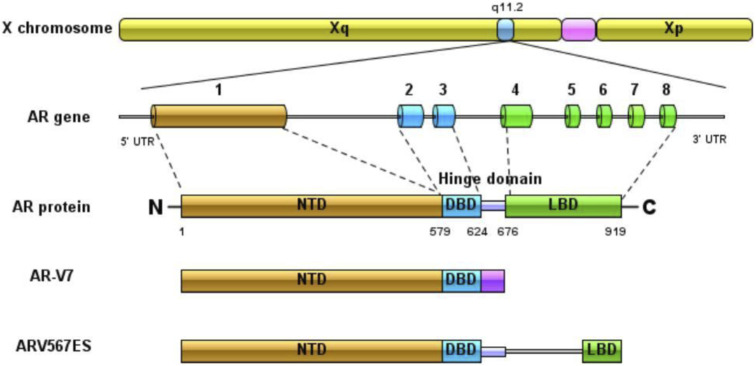
The transcriptional structure of full-length AR and major splicing variants AR-V7 and ARV567ES.

AR, also named NR3C4, is an important member of the nuclear receptor superfamily. Its full-length gene transcript contains eight exons, which synthesize three major functional protein domains with individual functions (Roy et al., 2001; Yu et al., 2020). The N-terminal domain (NTD) is encoded by exon 1, the DNA binding domain (DBD) is encoded by exons 2 and 3, and exons 4 to 8 encode the ligand-binding domain (LBD), which is connected to DBD through the flexure hinge region. The color boxes in Figure 2 represent individual protein domains including NTD, DBD, hinge, and LBD. All these domains are crucial for AR function. Recently, by using cryogenic electron microscopy (Cryo-EM), Yu et al. resolved the three-dimensional (3-D) structure of full-length AR protein (AR-FL), and observed the structural interaction of AR protein and the androgen responsive elements (ARE) binding motif in the promoter of target gene (Yu, et al., 2020).

AR co-regulators, recruited by the individual domains of AR protein, modify the epigenetic conditions around the ARE binding motifs in the chromatin, thereby affecting the transcriptional activity of AR. Compelling evidence has highlighted the central roles of chromatin structure and histone posttranslational modifications (PTMs) in determining the spectrum of genes regulated by AR and other transcription factors (Dehm and Tindall, 2007; Cai et al., 2013). Multiple histone modifying enzymes, together with demethylation enzymes directly regulate AR expression and its transcriptional activity (Leader et al., 2006). Additionally, non-coding RNAs, including long non-coding RNAs (lncRNAs) and micro RNAs (miRNAs), directly or indirectly modulate the epigenetic status to regulate AR-regulated gene transcription. The dysregulation of gene transcription contributes to PCa progression (Gao and Alumkal, 2010).

**FIGURE 3:**
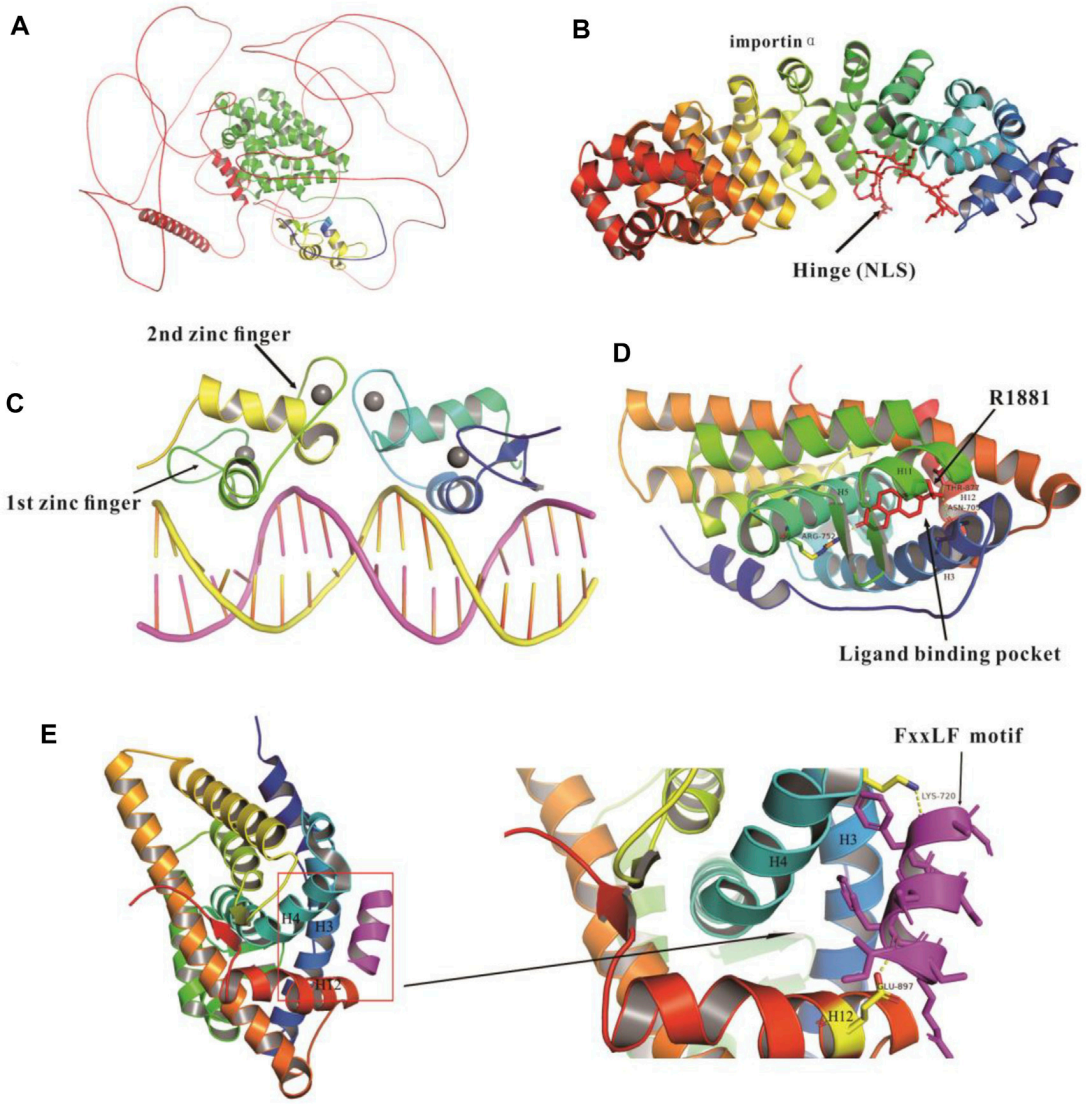
Crystal structures of full length, and domains of DBD, hinge and LBD of AR protein. **(A)** Crystal structure of full-length AR (Uniport: P10275, AlphaFold2). NTD, (amino acid residue 1-555) (red), DNA binding domain (DBD, amino acid residue 556-620) (yellow), hinge domain (amino acid residue 617-668) (blue); hinge and ligand binding domain (hinge-LBD, Amino acid residues 617-918) (green); LBD (amino acid residues 669-919) and AR NLS regions (amino acid residues 617-635) (color gradient). **(B)** Crystal structure of hinge domain and NLS regions (amino acid 621-635) (red) complexed with importin-α (PDB: 3BTR). **(C)** Crystal structure of DBD (PDB: 1R4I). **(D)** Crystallographic structure of protein complex containing LBD and a synthetic androgen metrexone R1881 (PDB: 1E3G), the ligand binding pocket surrounded by the N-terminus of H3, H5, H11 and H12. **(E)** Structure of AR LBD complex with FxxLF motif peptide (purple), the figure on the right shows the interface between AR LBD and FxxLF motif (PDB:1T7R). Hydrogen bonds are shown in dotted yellow lines.

The crystal structures of AR-FL, and the domains of hinge/DBD/LBD have been resolved (Figure 3), but that of NTD (AF-1), the strongest active domain has not been resolved yet.

### Co-Regulators Recruited by the N-Terminal Domain Domain of Androgen Receptor

The NTD of AR protein, a relatively long and flexible domain, accounts for more than 60% of AR protein, but the crystal structure of the NTD of AR protein has not been resolved yet. Within the NTD is activation function 1 (AF-1), which is essential for AR transactivational activity (Jenster et al., 1995; Dehm and Tindall, 2007). Co-activators of the p160 family such as SRC1 (steroid receptor coactivator 1) have been reported to directly bind and activate AF-1. The AF-1 contains two large domains TAU1 (amino acids 101-370) and TAU5 (amino acids 360-485), which are indispensable for receptor-dependent transactivation (Dehm and Tindall, 2007; Jenster, et al., 1995). Integrity of TAU-5 is a prerequisite for p160 co-activators recruitment by AR (Callewaert et al., 2006). In addition, the Cryo-EM decoded the 3-D structure of the protein complex containing DNA-bound full-length AR and its key co-activators SRC3 and p300, and identified the NTD as the primary site for the recruitment of co-activators (Yu, et al., 2020). HATs P300 and P300/CREB binding protein (CBP) induce AR and histone acetylation, and recruit proteins containing bromodomains such as BRD4, which promote the proliferation, migration and invasion of PCa cells (Gao and Alumkal, 2010; Belkina and Denis, 2012).

### Co-Regulators Recruited by the DNA Binding Domain/Hinge Domain of Androgen Receptor

The DBD/hinge domain of AR protein is the most conserved region and is composed of two zinc fingers based on the crystal structure of the DBD (Figure3) (Shaffer et al., 2004). Its functions include AR nuclear localization, dimerization, mediating AR DNA recognition and binding to the AREs (Dehm and Tindall, 2007). The first zinc finger is the P-box, by which AR binds to the nucleotide bases of the DNA major groove, responsible for sequence-specific DNA recognition. The second zinc finger is the D-box, including the AR DBD and carboxy-terminal extension (CTE), and this zinc finger is the AR dimerization interface, recapitulating the selectivity and specificity of the DNA binding site and inducing the dimerization of full-length receptor (Verrijdt et al., 2003; Dehm and Tindall, 2007). AR-DBD binds to the AREs in the prostate specific antigen (PSA) gene enhancers and induces the dimerization of AR DBD from head to head, which may be an appropriate model to explain androgen specificity (Tan et al., 2015). AR regulates the transmembrane protease serine 2 (TMPRSS2) gene by binding to the ARE sites in the promoter, which results in the abnormal overexpression of such ETS family oncogenes as ERG and ETV1. The fusion genes of TMPRSS2 and ETS oncogenes are associated with aggressive lesions, poor prognosis, and early-onset PCa (Tomlins et al., 2006; Tomlins et al., 2005; Verrijdt, et al., 2003). In addition, chromatin modifying enzymes, such as JMJD2C and LSD1, induce chromatin remodeling and modify AR binding to ARE sites to regulate gene transcription (Gao and Alumkal, 2010). Pioneer factors, a special class of TFs including FOXO and GATA2, bind to the compacted chromatin and initiate the recruitment of other TFs, such as ER or AR to access their DNA target sites to regulate gene transcriptional activation (Jozwik and Carroll, 2012).

**FIGURE 4:**
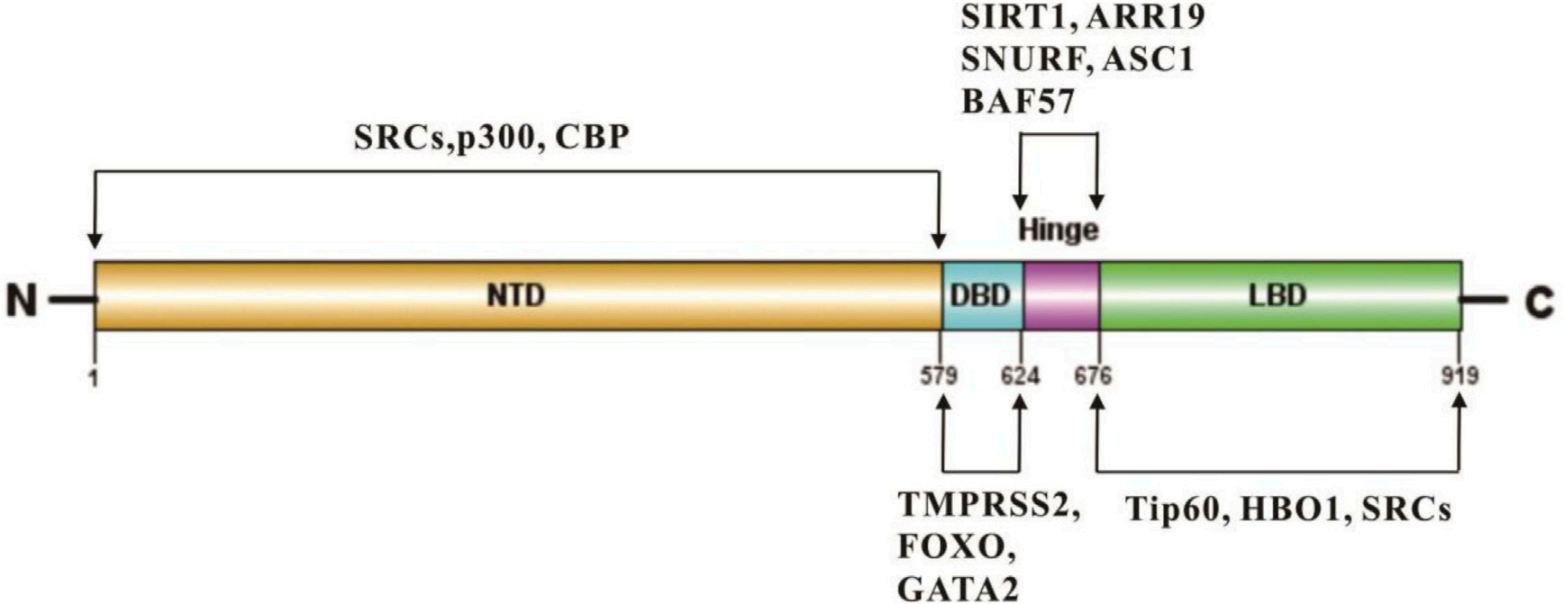
Representative epigenetic regulators bind to multiple domains of AR protein.

The hinge region is important for the subcellular distribution of nuclear receptors (Jenster et al., 1993; Zhou et al., 1994). Nuclear localization signals (NLS) located at the intersection of the DBD and hinge region of AR could regulate the nuclear import of nuclear receptors. The crystal structure of importin-α and AR shows that importin-α mediates the nuclear import of AR, and the residues from the major NLS 629-RKLKKL-634 contribute to importin-α binding to AR, and promote the AR transcriptional activity (Clinckemalie et al., 2012; Jenster, et al., 1993). Additionally, the hinge region of AR regulates the epigenetic pathway, especially the acetylation of its 629-RKLKKL-634 motif, which plays key roles in DNA binding, co-activator recruitment and the N/C interaction, and is a target site of acetylation, methylation and ubiquitination (Clinckemalie, et al., 2012; Fu et al., 2006). Multiple proteins, such as SNURF, ASC-1 and BAF57, work as co-activators of AR (Moilanen et al., 1998; Lee et al., 2002; Link et al., 2008), while others work as co-repressors including ARR19 and NcoR by binding to the hinge region Figure 4 (Wang H. et al., 2001; Jeong et al., 2004). In addition, SIRT1 induces the deacetylation of AR at K630, and thereby inhibits AR activity and the growth of PCa (Clinckemalie, et al., 2012).

### Co-Regulators Recruited by the Ligand-Binding Domain Domain of Androgen Receptor

The ligand-dependent functional binding domain (AF2) in glucocorticoid receptor (GR) or estrogen receptor (ER) shows strong transcriptional activity (Jenster et al., 1992; Yu, et al., 2020). However, the binding of ligand to AR-LBD initiates significant conformational changes, and forms a hydrophobic groove, which serves as a docking site for a LXXLL motif present in co-regulators, such as SRCs (Heery et al., 1997). The LBD contains the activation function 2 (AF-2, ∼250 amino acids) and mediates the interaction between AR and HSPs (Eftekharzadeh, et al., 2019; He et al., 2000), which prevents AR protein from degradation without androgen stimulation. AF-2 not only is the docking site of coactivators, but also promotes the N/C interaction, the recruitment of coactivators and AR transcriptional activation (Dehm and Tindall, 2007; Klokk et al., 2007). LBD interacts with NTD or other specific co-factors through the FXXLF motif to regulate gene transcription (Dubbink et al., 2006; Messner et al., 2020). The first crystallographic structure of AR-LBD-bound by the synthetic androgen metrexone R1881 has been resolved (Figure 3D) (Matias et al., 2000). R1881, by binding to LBD via hydrogen bonds, thus serves as the AR LBD agonist. LBD is the major binding site of anti-androgen drugs, but drug resistance occurs due to AR mutation and amplification.

The absence of LBD domain in splice variant AR forms (AR-Vs) constitutively sustains active AR signaling after anti-androgen drug treatment (Zhu and Luo, 2020).

AR-V7, a truncated AR splicing variant 7 without LBD, is highly active in CRPC, and promotes abiraterone and enzalutamide resistance (Nakazawa et al., 2014; Aurilio et al., 2020). Its mRNA retains the first three typical exons, and then connects with a mutation-specific recessive exon 3 (CE3). The splicing variation results from the early translation termination of 16 mutation-specific amino acids. In addition, ARv567ES retains the first 4 exons, connecting with the exon8, but misses the exons 5, 6 and 7.

## Epigenetic Control of Prostate Cancer

### DNA Methylation/Demethylation

DNA methylation is the most extensively studied epigenetic mechanism essential for gene transcription regulation. DNA methylation is primarily catalyzed by a family of DNA methyltransferases: DNMT1, DNMT3A and DNMT3B. DNMT1 is a major DNA methyltransferase for DNA methylation maintanace, while DNMT3A and DNMT3B, as *de novo* DNA methyltransferases, mainly initiate the DNA methylation (Okano et al., 1999). In PCa, many CpG islands display perturbed hypermethylation. Multiple key tumor suppressor genes such as p53, Rb1 and BRCA1, have undergone CpG islands hypermethylation in their promoter regions (Baylin, 2005). Aberrant DNA methylation has been linked to PCa initiation and progression (Nelson et al., 2009).

It has been reported that AR promoter is hypermethylated, which results in low AR expression in some of the PCa (Ruggero et al., 2018). DNMT1, cooperating with E2F1, down-regulates AR gene transcription in a methylation-independent manner (Valdez et al., 2011). Overexpression of DNMT1 induces DNA hypermethylation and represses the gene transcription of TMPRSS2, an AR target gene in AR-negative PCa cells (Chu et al., 2014). In contrast, DNMT1 knockdown leads to de-repression of endogenous AR in human normal prostate epithelial cells (Valdez, et al., 2011).

DNA methylation patterns are largely erased and then re-established between generations of mammals. Ten-eleven translocation proteins (TET1-3) mediate the oxidation of 5-methylcytosine (5mC) to 5-hydroxymethylcytosine (5hmC), 5-formylcytosine (5fC), and 5-carboxycytosine (5caC) (Tahiliani et al., 2009; He et al., 2011; Ito et al., 2011). And then, the oxidized forms are directly excised by DNA glycosylases such as thymine DNA glycosylase (TDG) (He, et al., 2011). 5hmC, the major oxidized form of 5mC, is profoundly reduced in human cancers, including PCa. DNA hypomethylation is often observed in the metastasized PCa. Hypomethylation in the promoter region of oncogenes leads to activate transcription and promotes cell proliferation. For example, the reduced genome-wide methylation weakens the stability of chromatin and promotes the expression of proto-oncogene MYC and RAS, thereby promoting PCa invasion and metastasis (Gurel et al., 2008; Albany et al., 2011).

### Histone Modifications

#### Histone Methylation

Histone modifications play vital roles in cancer progression (Ruggero, et al., 2018). Methyltransferases (KMTs) and demethylases (KDMs) are important regulators of histone methylation and gene transcription (Table 1) (Coffey et al., 2013; Askew et al., 2017). AR-dependent gene transcription depends on both histone methyltransferase and demethylase activities. The protein arginine methyltransferase (PRMT-4), more commonly known as co-activator-associated arginine methyltransferase 1 (CARM-1), by interacting with SRC co-activators, is essential for specific arginine methylation on histones H3. CARM-1 promotes AR-mediated transactivation completely depending on the presence of SRC proteins. Due to its indirect recruitment to AR, CARM-1 has been classified as a secondary co-activator (Chen et al., 1999). Additionally, PRMT1 is recruited to the AR transcriptional complex and promotes AR-dependent gene expression through SRC proteins (Wang Q. et al., 2001). The methyltransferase G9a is predominantly related to transcriptional repression by regulating the methylation lysine 9 on histone 3 (H3K9). However, G9a functions as a co-activator by synergistically working with TIF-2 and CARM-1 (Lee et al., 2006). LSD1 removes the methyl groups from dimethylated or monomethylated Lys 4 or Lys 9 on histone H3, thereby acting as a co-activator or co-suppressor of AR (Lee, et al., 2006). JMJD2C cooperates with LSD1 to demethylate the repressively trimethylated H3K9 during gene activation by AR (Schulz and Hoffmann, 2009). The histone demethylase JHDM2A cooperates with AR, and induces H3K9 demethylation as well as transcriptional activation (Yamane et al., 2006).

**TABLE 1:** An overview of coregulators that modulate AR activity.

Coregulators	Function	Binding region	References
Histone methylation			
CARM-1	co-activator	indirect	Majumder et al. (2006)
PRMT-1	co-activator	indirect	Wang, et al. (2001a)
PRMT-5	co-activator	indirect	Hosohata et al. (2003)
G9a	co-activator	indirect	Lee, et al. (2006)
NSD1	co-activator	DBD-LBD	Rayasam et al. (2003)
LSD1	co-activator	NTD,DBD,LBD	Metzger et al. (2005); Wissmann et al. (2007)
JARID1B	co-activator	direct	Xiang et al. (2007)
JHDM2A	co-activator	direct	Yamane, et al. (2006)
JMJD2C	co-activator	direct	Wissmann, et al. (2007)
JMJD2B	co-activator	direct	Coffey, et al. (2013)
PHF8	co-activator	direct	Tong et al. (2016)
Histone acetylation			
SRC1-3	co-activator	direct	Alen et al. (1999); Ding et al. (1998); Huang et al. (2003); Ma et al. (1999)
Tip60	co-activator	LBD	(Gaughan et al. (2001); Gaughan, et al. (2002)
p300	co-activator	Indirect/direct	Fu et al. (2000)
CBP	co-activator	Indirect/direct	Aarnisalo et al. (1998)
P/CAF	co-activator	Indirect/direct	Fu et al. (2003); Fu, et al. (2000)
SIRT1	co-repressors	hinge	Fu, et al. (2006)
HBO1	co-repressors	DBD and LBD	Sharma et al. (2000)
HDAC1	co-repressors	DBD-LBD	Gaughan, et al. (2002)
HDAC2	co-repressors	direct	Chng et al. (2012)
HDAC7	co-repressors	indirect	Karvonen et al. (2006)

#### Histone Acetylation

Histone acetylation is generally associated with active transcription, regulated by HATs and HDACs (Table 1) (Mathis et al., 1978; Grunstein, 1997; Abbas and Gupta, 2008).

Tip60, a histone acetyltransferase abnormally overexpressing in CRPC tissues, directly induces the acetylation of H2A, H3 and H4 through the HAT region at the C-terminal, and directly induces AR acetylation through the activation of the intrinsic acetyltransferases (Gaughan et al., 2002). Histone acetyltransferase P300 and PCAF (p300/CBP-associating factor) induce the acetylation of three lysine residues of AR (Lys-630 and Lys-632 and Lys-633), thereby promoting the intrinsic transcriptional activity of AR. The acetylation of AR may be reversed by HDACs including Sirtuin 1, thereby reducing the transcriptional activity of AR (Fu, et al., 2006; Lavery and Bevan, 2011). Downregulation of SIRT2 deacetylase elevated the acetylated Lys 18 on H3, which decreased the sensitivity to ADT (Damodaran et al., 2017). Altogether, HATs and HDACs as co-regulators have been involved in the AR-mediated gene transcription.

### Noncoding RNAs

**FIGURE 5:**
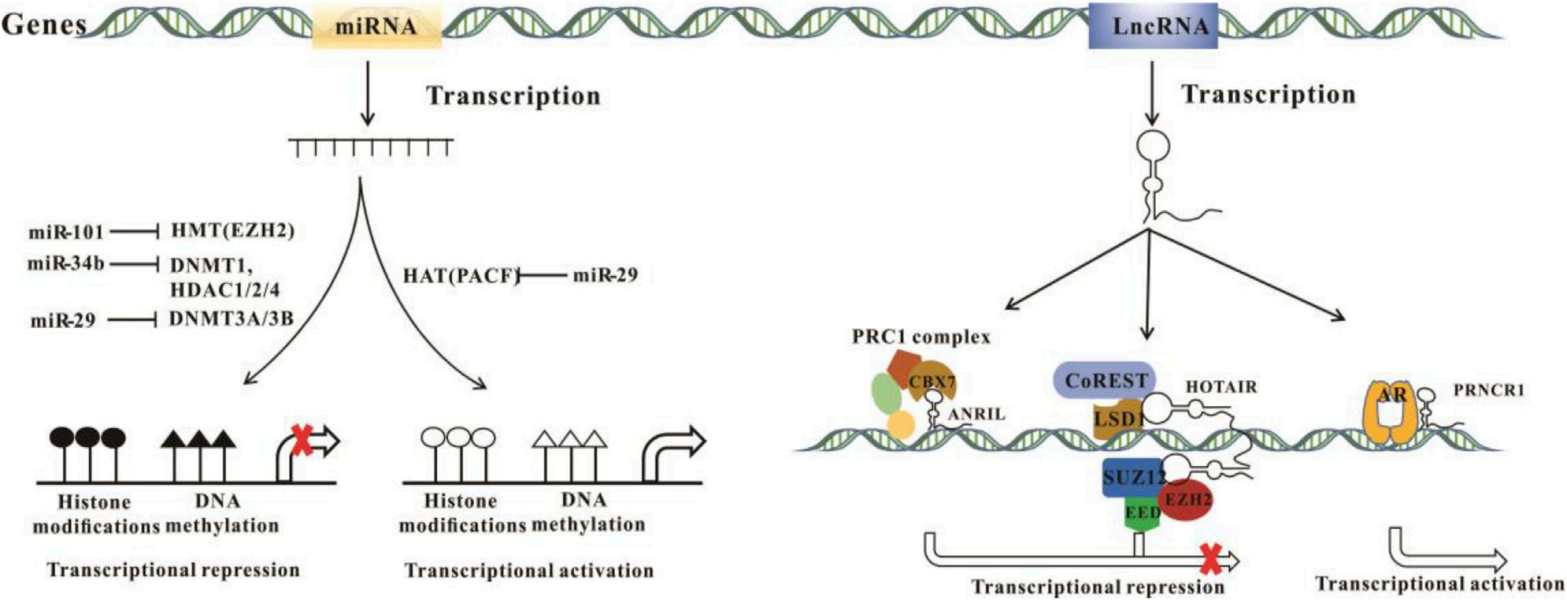
The mechanisms of miRNA and lncRNA in epigenetic regulation.

Non-coding RNAs (ncRNAs) refer to RNAs that are not translated into proteins. According to their length, ncRNAs are divided into two categories: small ncRNAs are transcripts with a length of 18–200 nt, and long ncRNAs are those more than 200 nt. NcRNAs participate in epigenetic regulation directly or indirectly (Figure 5).

#### Long Non-Coding RNAs

LncRNAs primarily interact with mRNA, DNA, protein, and miRNA and consequently regulate gene expression through epigenetic mechanisms. LncRNAs repress gene transcription in collaboration with chromatin remodeling complexes or histone modifying enzymes. The polycomb repressive complexes PRC1 and PRC2 as the most common protein partners of lncRNAs, result in chromatin compaction. PRC2 is mainly composed of SUZ12, EED and EZH2. Among them, histone methyltransferase EZH2, responsible for H3K27 methylation, leads to gene silencing (Wei et al., 2017). Multiple lncRNAs bind to PRC2 and function in a PRC2-dependent manner. For example, HOTAIR interacts with PRC2 via its 5′ domain and regulates the epigenetic repression of PRC2 target genes (Rinn et al., 2007). Furthermore, HOTAIR contains another binding domain at the 3’ end for LSD1/CoREST, a histone deacetylase complex that facilitates transcriptional repression (Tsai et al., 2010). Similarly, in addition to PRC2 proteins, ANRIL also binds CBX7, a component of PRC1, then recruits PRC1 complexe to the INK4A/ARF locus to inhibit transcription (Yap et al., 2010).

LncRNAs have been involved in transcription activation. PRNCR1 and PCGEM1 bind successively to AR and enhance AR-mediated gene transcription in a ligand-dependent or independent manner (Yang et al., 2013). Linc00675 promotes AR signaling and PCa progression by two ways. Linc00675 binds to the NTD domain of AR protein, and competitively inhibits the binding of MDM2, thereby inhibits AR protein degradation through ubiquitin-proteasome pathways. On the other hand, linc00675 promotes AR signaling by binding and stabilizing GATA2 mRNA (Yao et al., 2020). Table 2 summarizes the lncRNAs involved in PCa.

**TABLE 2:** Epigenetic regulatory functions of lncRNAs.

LncRNA	Expression in PCa	Function and mechanism	Relationship with AR	Clinical relevance	Ref
BLACAT1	↓	Downregulated by histone deacetylation and DNA methylation, inhibited the growth of PCa cells	—	Diagnostic biomarker	Li et al. (2020)
LINC00673	↑	Increases methylation of kl4 promoter region, promote the development of PCa	—	Therapeutic target	Jiang et al. (2020)
NEAT1	↑	Promotes the transcription of target gene promoter to drive cancer development	-	Prognostic/predictive biomarker	Chakravarty et al. (2014)
ANRIL	↑	Participates directly in epigenetic transcriptional repression and regulates gene silencing	—	Therapeutic target	Yap et al. (2010)
HOTAIR	↑	Binds with EZH2 to inhibit tumor suppressor gene	Upregulate AR	Prognostic/predictive biomarker	Ling et al. (2017); Zhang et al. (2015)
HOTTIP	↑	Induces chromatin modification to regulate gene expression	—	Prognostic biomarker	Lee et al. (2019)
DANCR	↑	DANCR and EZH2 jointly inhibited TIMP2/3	Downregulated by AR	Therapeutic target	Jia et al. (2016)
PVT1	↑	Induces promoter methylation of miRNA- 146a	—	Prognostic/predictive biomarker	Liu et al. (2016)
SOCS2-AS1	↑	Regulates AR target gene and promotes androgen signal transduction	Upregulated by AR	Therapeutic target	Misawa et al. (2016)
ZEB1-AS1	↑	Interacts and recruits MLL1/induce H3K4me3/up-regulate	—	Therapeutic target	Su et al. (2017)
		ZEB1			
CTBP1-AS	↑	Regulates epigenetic cancer related genes to promote CRPC	Upregulated by AR	Therapeutic target	Takayama et al. (2013)
HOXC-AS1	↑	Interacts with U2AF2 and promotes AR mRNA splicing	Upregulated by AR	Therapeutic target	Takayama et al. (2020)
PRKAG2-AS1	↑	Interacts with U2AF2 and promotes AR mRNA splicing	Upregulate AR	Therapeutic target	Takayama, et al. (2020)
ARLNC1	↑	ARLNC1 is not only induced by AR protein, but also stabilized by RNA-RNA interaction	Upregulated by AR/Upregulate AR	Therapeutic target	Zhang et al. (2018)
LINC00844	↓	Enhances global expression of androgen regulated genes	Upregulated by AR	Therapeutic target	Lingadahalli et al. (2018)
MEG3	↓	Promotes H3K27 trimethylation of EN2 by binding with EZH2, thus inhibiting the occurrence of PCa	—	Therapeutic target	Zhou et al. (2020)
LINC00675	↑	Regulates the binding between AR and MDM2, and activates AR signaling pathway by interacting with GATA2 mRNA	Upregulate AR	Therapeutic target	Yao, et al. (2020)
TMPO-AS1	↑	Promotes the progression and migration of cancer cell cycle, reduce the apoptosis of PCa cells	Downregulated by AR	Diagnostic/prognostic biomarker	Huang et al. (2018)
PlncRNA-1	↑	PlncRNA-1 antagonizes the post transcriptional regulation of AR by miR-34c and miR-297	Upregulate AR	Therapeutic target	Fang et al. (2016)
DRAIC	↓	FoxA1 and NKX3-1 were recruited into the DRAIC site of AR to induce DRAIC	Downregulated by AR	Prognostic/predictive biomarker	Sakurai et al. (2015)
PCAT29	↓	Located downstream of DRAIC and regulated by AR, FoxA1 and NKX3-1	Downregulated by AR	Prognostic/predictive biomarker	Sakurai, et al. (2015)
PCAT1	↑	Interacts with AR and LSD1 to promote prostate cancer cell growth	Upregulated by AR	Therapeutic target	(Guo et al. (2016); Shang et al. (2019)
HOXD-AS1	↑	Interacts directly with the promoter region of the target genes and mediates H3k4me3 activation transcription by binding with WDR5	—	Therapeutic target	Gu et al. (2017)
PCGEM1	↑	Interacts with AR protein and enhances its transactivation	Upregulate AR	Diagnostic/Prognostic biomarker	Parolia et al. (2015)
SChLAP1	↑	Antagonizing SWI/SNF chromatin modification complex and promoting the invasiveness of PCa	-	prognostic/predictive biomarker	Mehra et al. (2016)
GAS5	↓	Interaction between GAS5 and E2F1 activates cell cycle regulator	—	Therapeutic target	Luo et al. (2017)
LBCS	↓	Interacts with hnRNPK and AR mRNA to inhibit AR translation	Downregulate AR	Therapeutic target	Gu et al. (2019)
MALAT1	↑	Silencing MALAT1 inactivated AR signaling by sponging miR-320b, and inhibited proliferation and cell cycle progression	Upregulate AR	Diagnostic/prognostic biomarker	Dai et al. (2019)
				Therapeutic target	
PRNCR1	↑	Interacts with AR protein and enhances its transactivation	Upregulate AR	—	Yang et al. (2013)
SARCC	—	SARCC inhibited AR function, inhibited miR-143-3p and its downstream Akt, MMP-13, K-ras and p-ERK signaling	Downregulate AR	—	Zhai et al. (2017)

#### Micro RNAs

miRNAs, by binding to the 3′ untranslated regions (UTR) of mRNA, repress gene expression. Aberrant miRNAs may be the outcome of epigenetic dysregulation, including CpG islands hypermethylation of promoter (Esteller, 2011). The methylation sensitive E2F transcription factors have been involved in miRNA regulation (Emmrich and Putzer, 2010). For example, the hypermethylation of CpG sites in promoters of miR-200c and miR-141 repress their expression. Conversely, upregulation of miR-34b repressed the expression of DNMTs and HDACs by directly targeting the 3′UTR of their mRNA, and partially induced demethylation and active chromatin modification (Majid et al., 2013).

miRNAs have emerged as the key regulators in diverse biological processes. miR-200 promotes epithelial mesenchymal transformation (EMT) by decreasing ZEB1 and ZEB2 (Park et al., 2008). miR-135a overexpression reduces PCa cell invasion and migration *in vivo* and *in vitro* by downregulating ROCK1 and ROCK2 (Kroiss et al., 2015).

The interaction between AR signaling and miRNAs contributes to the progression of PCa. AR binds to miR-21 promoter and increases its expression (Ribas et al., 2009). The upregulation of miR-21 inhibits the expression of transforming growth factor β receptor II (TGFBR2) by binding to its 3′UTR, thus attenuating TGFβ-mediated smad2/3 activation and cell growth inhibition in PCa (Mishra et al., 2014). In addition, AR directly binds to the AR binding site of the miR-135a and activates its transcription (Kroiss, et al., 2015).

Conversely, miRNAs repress AR gene expression. miR-31 directly binds to the mRNA of AR or other cell cycle regulatory genes and inhibit the proliferation of PCa cells (Lin et al., 2013). In addition, miR-34 and AR regulate each other in a feedback loop. miR-34 directly targets the 3’ UTR of AR mRNA and inhibits AR gene expression. In contrast, miR-34 is a direct target of p53 (He et al., 2007). AR acts upstream of p53 in PCa and activates the gene transcription of miR-34 in p53-dependent manner (Rokhlin et al., 2008).

In addition to direct regulation, miRNAs also indirectly regulate the gene expression and transcriptonal activity of AR. For example, miR-141 targets the 3′ UTR of Orphan receptor small heterodimer partner (SHP) mRNA, resulting in the translation inhibition and RNA degradation. SHP is a common suppressor of AR, which inhibits AR transcriptional activity. miR-141 is commonly elevated in PCa, thus indirectly promotes AR transcriptional activity (Xiao et al., 2012). In addition, MYC activates AR transcription by binding to the promoter. miR-let-7c inhibits AR gene expression and activity via targeting the 3’ UTR of *MYC*, which results in the degradation of *MYC* mRNA through RISC complex (Nadiminty et al., 2012). Table 3 summarizes the miRNAs involved in PCa.

**TABLE 3:** miRNAs as epigenetic regulators.

miRNA	Expression in PCa	Function and mechanism	Relationship with AR	Clinical relevance	Ref
miR-130a	↓	miR-130a promoter methylation leads to its down-regulation, which promotes the malignant phenotype of PCa	—	Therapeutic target	Ramalho-Carvalho et al. (2017)
miR-23b	↓	Targets Src kinase and Akt, and its expression is regulated by promoter methylation	—	Diagnostic/prognostic biomarker/therapeutic target	Majid et al. (2012)
miR-193b	↓	Hypermethylation of the miR-193b promoter releases the inhibition of oncogenes	—	Diagnostic/prognostic biomarker	Mazzu et al. (2019)
miR-200	↓	Inhibits EMT	—	prognostic biomarker	Lynch et al. (2016)
miR-129-2	↓	Promoter hypermethylation in prostate cancer	—	Diagnostic/prognostic biomarker	Mazzu, et al. (2019)
miR-31	↓	Targeting androgen receptor and other cell cycle regulators to inhibit the growth of PCa	Downregulated by AR	Therapeutic target	Lin et al. (2013)
miR-205	↓	Targeting MED1 inhibits the expression of MED1 and the progression of PCa	—	Therapeutic target	Hulf et al. (2013)
miR-34	↓	miR-34 promoter hypermethylation inhibited by AR-p53pathway	Downregulated by AR	Therapeutic target	Rokhlin, et al. (2008)
miR-338-5p/miR-421	↓	The expression was affected by DNA methylation and EZH2	—	Therapeutic target	Bhatia et al. (2019)
miR-21	↑	AR up-regulates miR-21and increases the proliferation of PCa cells	Upregulated by AR	Therapeutic target	Ribas, et al. (2009)
miR-135a	↓	AR binds to the AR site of miR-135a and inhibits PCa invasion and migration	Upregulated by AR	Prognostic biomarker	Kroiss, et al. (2015)
miR-101	↓	Negative regulation of EZH2 affects PCa progression	Upregulated by AR	Therapeutic target	Cao et al. (2010)
miR-320a	↓	Reduces AR mRNA and protein levels and inhibits proliferation of PCa	Downegulate AR	Therapeutic target	Sato et al. (2016)
miR-421	↓	Direct targeting NRAS, PRAME, CUL4B and pfkfb2 inhibits tumor development	Downregulated by AR	Therapeutic target	Meng et al. (2016)
Let-7c	↓	Regulates AR expression through a transcriptional mechanism involving in Myc	Downregulate AR	Therapeutic target	Nadiminty et al. (2012)

Let-7d	↓	Androgen promotes PCA by regulates pbx3 expression through let-7d	Upregulated by AR	Therapeutic target	Ramberg et al. (2011)
miR-148a	↑	Increased expression of miR-148a inhibits its target gene CAND1 and promotes the progression of PCa	Upregulated by AR	Therapeutic target	Murata et al. (2010)
miR-29	↑	Inhibits the expression of tet2 to promotes the progression of PCa	Upregulated by AR	Therapeutic target	Takayama et al. (2015)
miR-488*	↓	Inhibits the expression of AR by binding to the 3′UTR site of AR mRNA	Downregulate AR	Therapeutic target	Sikand et al. (2011)
miR-145	↓	miR-145 was down regulated by promoter methylation, resulting in down-regulation of proapoptotic gene TNFSF10	—	Therapeutic target	Zaman et al. (2010)
miR-197-3p	↓	miR-197-3p/VDAC1/AKT/β-catenin signaling axis regulates PCa cell growth	Upregulate AR	Therapeutic target	Fletcher et al. (2019); Huang et al. (2020)
miR-346	↑	CircDDX17 competes with miR-346 to act as a oncogenic role and up-regulate LHPP	Upregulate AR	Prognostic biomarker/Therapeutic target	Fletcher et al. (2019); Lin et al. (2020)
miR-361-3p	↓	Inhibits the expression of ARv7 and MKNK2 and enhances the sensitivity of ENZ	Downregulate AR	Therapeutic target	Fletcher et al. (2019); Liu et al. (2020)
miR-541-3p	↓	Inhibits the HSP27 expression and downregulates β-catenin	—	Therapeutic target	He et al. (2021)
miR-141	↑	Inhibits SHP and indirectly upregulates AR	Upregulate AR	Therapeutic target	Xiao, et al. (2012)

#### Non-Coding RNAs and Liquid-Liquid Phase Separation

Liquid liquid phase separation (LLPS) is a biological phenomenon, which means that components with similar properties form droplets and condense in cells. The intracellular components form a reaction chamber through phase separation, so as to establish an environment in which multiple biological reactions occur at the same time (Shin and Brangwynne, 2017). NcRNAs play an important regulatory role in phase separation. NcRNAs contain different RNA domains, which are recognized and bound by specific RNA binding proteins (RBPs), which put together to form a dynamic network called phase separation droplets (Nozawa et al., 2020).

Modifications in LLPS result in a series of epigenetic disorders, then further promote tumorigenesis (Erdel and Rippe, 2018; Nozawa, et al., 2020). Biomolecular condensates are formed through LLPS, which separate and concentrate biomolecules with different physical and chemical properties. Small molecule anti-cancer drugs may be concentrated, and then selectively distributed into the condensate, so as to modify its pharmacodynamic characteristics (Klein et al., 2020). Such oncogenes as RAS and MYC, are considered as undruggable targets due to the lack of protein pockets, can be targeted by the condensates containing small molecules anti-cancer drugs (Dang et al., 2017). Transcriptional co-activators such as BRD4 and MED1 have been shown to form foci at superenhancer (SE) sites, showing the nature of liquid condensate. AR interacts with BRD4 and MED1, and forms foci in AR-positive PCa cells. AR rich foci formation show the characteristics of liquid-like condensate, which may be phase separated condensate. However, foci formation requires the full-length structure of AR, while the truncated ARs are not enough to induce lesions (Zhang et al., 2021). AR NTD containing multiple potential Speckle-type POZ protein (SPOP) interaction motifs ARE sufficient to drive the LLPS of AR (Bouchard et al., 2018). AR NTD form droplets at high concentration *in vitro*, and SPOP strongly promotes the formation of droplets (Bouchard, et al., 2018). In addition, studies have suggested that AR DBD is the main LLPS driver, which is the smallest region separated from RNA. Without a small amount of RNA, DBD could not drive LLPS alone, and only unstructured RNA make DBD drive LLPS (Ahmed et al., 2021). These studies show that AR drive LLPS to form phase condensate by different mechanisms. Since the unfolding mechanism of AR LLPS may be the key to cancer progression, it is very important to further study the mechanism details of AR LLPS.

Currently, the study of phase separation is far from mature, and many questions need be addressed. For example, how do ncRNAs regulate phase separation? how do ncRNAs regulate the pharmacodynamics of drugs through aggregates? and how do ncRNAs find the phase separation of more proteins? The resolution of these questions is critical to clarify the roles of ncRNAs in cancer progression, and further to identify ncRNAs as novel anti-cancer drug targets.

## Epigenetic Regulators as Prostate Cancer Diagnostic Markers

Current methods of PCa diagnosis mainly test the levels of serum PSA. Nevertheless, PSA specificity testing alone leads to false positive results and unnecessary biopsies. Epigenetic regulators may become sensitive and specific diagnostic biomarkers for early PCa detection due to their advantages of stability and availability in body fluids. Epigenetic regulator biomarkers may avoid unnecessary biopsies and reduce the economic burden of high-cost overtreatment (Smith-Palmer et al., 2019).

### Epigenetic Modifications of Key Genes as Biomarkers

Methylation of GSTP1 promoter is the most common epigenetic modification in PCa. GSTP1 promoter methylation was found in 90% of adenocarcinomas and 70% of high-grade and pre high-grade PCa niduses, but not in normal or hyperplastic prostatic epithelium (Nakayama et al., 2003). Hypermethylation of GSTP1 promoter can accurately distinguish whether a person has PCa or not, with a significant specificity of 89.5–100% and sensitivity of 21.4–86.3% in urine samples (Cairns et al., 2001). A PCa methylation assay (ProCaM™) assessing GSTP1, APC and RAR in urine has been validated in a multicenter prospective study in which urine samples from men with serum prostate PSA levels of 2.0–10.0 ng/ml were analyzed. This method can detect PCa more accurately than serum PSA (Baden et al., 2011).

Another kind of epigenetic modification that can distinguish normal tissue from PCa is histone modification. The levels of H4Ac, H3Ac, H3K4, H3k9me3, H3K9me2 and H3k4me1 in PCa are significantly decreased. H3K9me2 and H3Ac have almost 90% specificity and 80% sensitivity for distinguishing tumor tissue from non-malignant tissue (Santos et al., 2020).

### Non-Coding RNAs as a Prostate Cancer Diagnostic Biomarker

#### Long Non-Coding RNAs

PCa tissues show increased expression of MALAT-1 compared with normal and benign prostatic hyperplasia tissues (Ren et al., 2012). Downregulation of MALAT-1 inhibited the growth of PCa cells and resulted in cell cycle arrest. Circulating MALAT-1 fragments (MD minirna) were better than PSA for predicting prostate biopsy results, indicating that MALAT-1 is a potential biomarker for PCa diagnosis (Wang et al., 2014).

Progensa™ PCA3 (Gen-Probe Inc., San Diego, CA, United States) has been approved by the United States FDA for detecting the levels of PCA3 in the urine of men over 50 years of age. In most prostate tumors, the level of PCA3 is 60–100 times higher than in adjacent non-tumor tissues, and is not detected in other types of tumors. Compared with serum PSA, the combination of urine PCA3 and fusion gene TMPRSS2-ERG significantly improved the specificity of PCa diagnosis and reduced unnecessary prostate biopsy (Salagierski and Schalken, 2012).

*PCA3* is an ideal diagnostic marker, but is not a prognostic marker (Leyten et al., 2014). However, PCAT14 may be a diagnostic and prognostic biomarker, since it is highly expressed in non-malignant tumor tissues, while the absence of PCAT14 promotes proliferation and recurrence of cancer. Its expression inversely correlates with the degree of aggressiveness, and may be used as a conventional clinicopathologic risk factor for PCa prognosis. Expression of PCAT14 is correlated with better biochemical progression-free survival, metastasis-free survival, and PCa-specific survival (Shukla et al., 2016).

Another promising prognostic urine biomarker is lncRNA *SChLAP1*, which is readily detected in urine sediments by qPCR. The expression of SChLAP1 is specific to PCa and functions as independent risk factor for metastasis (Prensner et al., 2014).

#### Micro RNAs

miRNAs can be detected in serum, plasma and urine samples, and may become highly specific, stable and sensitive biomarkers for PCa diagnosis. Moya et al. reported that the levels of miR-98-5p, miR-152-3p, miR-326 and miR-4289 are higher in plasma samples of PCa patients than the healthy controls (Moya et al., 2019). The specificity and sensitivity of miR-17-3p and miR-1185-2-3p as a diagnostic tool is over 90% (Urabe et al., 2019). miR-182-5p was significantly upregulated in the tissues and plasma of PCa patients. miR-182-5p expression in PCa had an AUC in tissues of 0.81, a specificity in plasma of 77%, and an NPV of 99% (Bidarra et al., 2019).

## Epigenetic Regulators as Therapeutic Targets for Drug Development

Epigenetic drugs have shown great anti-cancer potential in preclinical studies and clinical trials through different pathways. Recently, BET inhibitors targeting BRD4, a newly identified epigenetic molecule, demonstrated significant anti-cancer effects (Mahajan et al., 2017).

JQ1, an inhibitor of BET, inhibits the binding of BRD4 to AR enhancers, and thereby represses AR signaling and AR-regulated gene transcription (Lochrin et al., 2014). However BET inhibitors did not show satisfactory survival benefit for PCa patients in the clinical trials. In CRPC, EZH2 functions as a co-activator of AR to regulate gene transcription. PI3K/AKT phosphorylates EZH2 at S21 and reduces the H3K27me3 activity of EZH2 (Xu et al., 2012). Phosphorylated EZH2 interacts with AR and methylates AR at the sites of lysine 630 and 632, which increase its transcriptional activity (Deb et al., 2013; Xu, et al., 2012). EZH2 inhibitors alone or in combination with other inhibitors, such as NF-κB inhibitors, have demonstrated great therapeutic potential for PCa (Wu et al., 2016; Jin et al., 2021). EZH2 or AR inhibitors alone did not demonstrate significant anti-cancer efficacy, and easily induced drug resistance due to the reciprocal feedback activation loop (Shankar et al., 2020). However, the combination of EZH2 and AR inhibitors synergistically inhibited the growth of CRPC (Kim et al., 2018; Shankar, et al., 2020).

The histone methyltransferase inhibitor (HMTi) 3-dezaneplanocin-A (DZNeP) showed significant antitumor efficacy by inhibiting the activity of multiple histone methyltransferases including EZH2 (Miranda et al., 2009). LSD1, as a key histone demethytransferase, is upregulated in CRPC, and is highly and positively correlated with the prognosis of CRPC patients. LSD1 inhibitors have been developed (Metzger, et al., 2005; Yang et al., 2018), and demonstrated significant anti-cancer efficacy by inhibiting cancer cell proliferation, differentiation, invasion and migration (Fang et al., 2019).

HDAC molecules regulate gene expression by altering chromatin structure and regulating the functions of non-histone proteins (Chao and Goodman, 2014). Several HDAC inhibitors (HDACi) have been developed, and demonstrated anti-cancer potential by inducing cell cycle arrest, apoptosis and autophagy, and inhibiting the production of reactive oxygen species (ROS) (Rana et al., 2020). Inhibition of HDAC6 results in the hyperacetylation of HSP90, the loss of ATP binding, and the dissociation and degradation of AR (Rosati et al., 2016).

Additionally, natural compounds like anacardic acid, from cashew nut shell liquid, and a polyisoprenylated benzophenone derivative from the fruit rind of garcinia indica, known as garcinol, inhibit the HAT activity of both p300 and PCAF (Balasubramanyam et al., 2004). Small molecule synthetic inhibitors such as the γ-butyrolactone MB-3 are cell-permeable GCN5 inhibitor, and a series of isothiazolones have been found to inhibit p300 and PCAF activity (Biel et al., 2005). The epigenetic targets of flavonoids include oncogenes and tumor suppressor genes. They are indirectly regulated by epigenetic enzymes such as DNMTs, HATs and HDACs. As natural epigenetic regulators, they have been used for chemoprevention, which is a promising and effective strategy to inhibit carcinogenesis and progression (Izzo et al., 2020). Bioactive molecules in the diet, such as the edible plant Celtis sagittata, demonstrated remarkable anti-cancer efficacy by regulating epigenetic signaling pathways and altering tumor-associated immune response. These compounds have anticancer functions in various cancer types by specifically inhibiting the activities of DNMTs and HDACs (Pradhan et al., 2019). In addition, many epigenetic drugs have been tested in various stages of clinical trials (Table 4).

**TABLE 4:** Development of epigenetic drugs.

Trial ID	Drug	Phase	Conditions	Status
			AR inhibitor	
NCT02987829	TRC253	1and2	mCRPC; PCa	Completed
NCT00326586	BMS-641988	1	PCa	Completed
NCT00103376	Velcade	2	PCa	Terminated
	LH-RH-Agonist			
	AR Antagonists			
NCT02826772	GT0918	1	mCRPC	Completed
NCT02972060	ODM-201	2	PCa	Recruiting
	ADT			
NCT03124433	Apalutamide	2	PCa	Completed
NCT01981122	Enzalutamide	2	Metastatic PCa	—
NCT04381832	Etrumadenant	1and2	mCRPC	Recruiting
	Zimberelimab			
	AB680 Enzalutamide Docetaxel			
NCT01251861	MK2206	—	PCa	Active, not recruiting
NCT04104893	Pembrolizumab	2	mCRPC	Recruiting
**BET protein inhibitors**				
NCT04471974	ZEN-3694	2	CRPC	Recruiting
	Enzalutamide		Metastatic PCa, adenocarcinoma	
			Metastatic prostate small cell carcinoma	
NCT02711956	ZEN003694	1and2	mCRPC	Completed
	Enzalutamide			
NCT02259114	Birabresib	1	NUT midline carcinoma, TNBC	Completed
			NSCLC	
			With rearranged ALK gene/fusion protein or KRAS mutation	
			CRPC	
			Pancreatic ductal sdenocarcinoma	
**PARP inhibitors**				
NCT01085422	ABT-888	1	PCa	Completed
	Temozolomide			
NCT02324998	Olaparib Degarelix	1	PCa	Completed
NCT04703920	Talazoparib	1	Metastatic breast cancer mCRPC	Recruiting
	Belinostat		Metastatic ovarian cancer	
NCT03040791	Nivolumab	2	PCa	Recruiting
NCT04030559	Niraparib	2	PCa	Recruiting
	Niraparib-Tosylate-Monohydrate			
NCT04336943	Olaparib	2	Recurrent PCa	Recruiting
			Prostate adenocarcinoma	
NCT03317392	Olaparib	1and2	CRPC	Recruiting
			Metastatic prostate adenocarcinoma	
NCT02893917	Cediranib	2	CRPC, metastatic malignant neoplasm in bone, metastatic PCa	Active, not recruiting
	Olaparib		Prostate adenocarcinoma with neuroendocrine differentiation, prostate small cell neuroendocrine carcinoma	
NCT04644068	AZD5305 Paclitaxel Carboplatin	1	Ovarian cancer, breast cancer, pancreatic cancer, PCa	Recruiting
NCT04821622	talazoparib plus enzalutamide Placebo plus enzalutamide	3	PCa	Recruiting
NCT02975934	Rucaparib Abiraterone acetate or Enzalutamide or Docetaxel	3	mCRPC	Recruiting
NCT01286987	Talazoparib	1	Advanced or recurrent solid tumors, SCLC	Completed
			Ovarian cancer, PCa, pancreas cancer	
NCT04824937	Telaglenast Talazoparib	2	metastatic PCa	Recruiting
NCT03787680	Olaparib	2	PCa	—
	AZD6738			
**EZH2 inhibitors**				
NCT04407741	SHR2554 + SHR1701	—	Solid tumor lymphoma	Recruiting
	SHR1701			
NCT03480646	CPI-1205 Enzalutamide Abiraterone/Prednisone	1and2	mCRPC	Active, not recruiting
**PRMT5 inhibitors**				
NCT03854227	PF-06939999	1	Advanced solid tumors, metastatic solid tumors	Recruiting
	Docetaxel			
NCT03573310	JNJ-64619178	1	Neoplasms, solid tumora Adult non-Hodgkin lymphoma myelodysplastic syndromes	Recruiting
**LSD1/KMD1A inhibitors**				
NCT02717884	Tranylcypromine all-trans retinoic acid	1and2	AML, myelodysplastic syndrome	Recruiting
	cytarabine			
NCT02034123	GSK2879552	1	SCLC	Terminated
NCT03895684	SP-2577	1	Advanced solid tumors	Recruiting
NCT04350463	CC-90011 Nivolumab	2	neoplasms	Recruiting
**HDAC inhibitors**				
NCT01075308	SB939	2	PCa	Completed
NCT00878436	Panobinostat	1and2	PCa prostatic neoplasms	Completed
	Bicalutamide			
NCT00330161	vorinostat	2	Recurrent PCa	Completed
			Stage IV PCa	
NCT00020579	entinostat	1	cancers	Completed
NCT00667862	Panobinostat	2	PCa	Completed
NCT00045006	vorinostat	1	cancers	Completed
**HMT inhibitor**				
NCT03460977	PF-06821497	1	mCRPC	Recruiting
**AR-Vs inhibitors**				
NCT02532114	Niclosamide	1	Castration levels of testosterone	Completed
	Enzalutamide		CRPC	
			Metastatic PCa	
			Recurrent PCa	
			Prostate sdenocarcinoma	
NCT02090114	Testosterone cypionate	2	PCa	Recruiting
	Testosterone Enanthate			
	Abiraterone acetate			
	Enzalutamide			
NCT02286921	Testosterone cypionate	2	mCRPC	Completed
	Enzalutamide			
	Testosterone Enanthate			
NCT03511664	177Lu-PSMA-617	3	PCa	Active, not recruiting
NCT02807805	Abiraterone Acetate	1b	Metastatic PCa	Recruiting
	Niclosamide		Recurrent PCa	
	Prednisone		—	
NCT02606123	EPI-506	1and2	Prostatic neoplasms genital neoplasms, male	Terminated
			genital diseases, male	
			prostatic diseases	
NCT04421222	EPI-7386	1	PCa	Recruiting
**CBP/p300 inhibitors**				
NCT03568656	CCS1477	1and2	mCRPC	Recruiting
	Abiraterone acetate		Advanced solid tumors	
	Enzalutamide		—	
**PLK1 inhibitors**				
NCT03414034	Onvansertib	2	mCRPC	Recruiting
	Abiraterone Prednisone			
**Immune checkpoint inhibitors**				
NCT02601014	Ipilimumab	2	PCa	Active, not Recruiting
	Nivolumab		Recurrent PCa	
	—		Stage IV prostate adenocarcinoma	
NCT02985957	Nivolumab Ipilimumab	2	PCa	Recruiting
	Cabazitaxel Prednisone			
**DNMTs inbitors**				
NCT01118741	Disulfiram	Not Applicable	PCa	Completed

## Conclusion

The androgen/AR signaling pathway is one of the most important drivers for PCa initiation and progression, as well as the transformation to CRPC. Therefore, the anti-androgen/AR drugs have become the first line therapy for PCa.

With a deeper understanding of PCa biology and epigenetics, additional epigenetic regulators critical for the development of PCa have been identified. Epigenetic regulators, individually or in combination with AR, promote PCa progression and have the potential to become novel PCa diagnostic markers and therapeutic targets. Although the development of epigenetic drugs is still in its infancy, undoubtedly, they will become increasingly important therapeutic tools for PCa, particularly for CRPC and neuroendocrine prostate cancer (NEPC) when used alone or in combination with anti-androgen/AR drugs.
